# 5-Methyl-*N*′-(3-nitrobenzylidene)isoxazole-4-carbohydrazide

**DOI:** 10.1107/S1600536809051733

**Published:** 2009-12-04

**Authors:** Yan-Xian Jin

**Affiliations:** a School of Pharmaceutical and Chemical Engineering, Taizhou University, Linhai 317000, People’s Republic of China

## Abstract

The mol­ecule of the title compound, C_12_H_10_N_4_O_4_, displays an *E* configuration about the C=N bond. The dihedral angle between the benzene and isoxazole rings is 1.36 (5)° and the mol­ecular conformation is stabilized by the an intra­molecular C—H⋯N hydrogen bond. In the crystal structure, centrosymmetrically related mol­ecules are connected by pairs of N—H⋯O hydrogen bonds into dimers, which are further linked into a three-dimensional network by inter­molecular C—H⋯O hydrogen bonds and by π⋯π stacking inter­actions involving adjacent benzene and isoxazole rings, with a centroid–centroid separation of 3.861 (3) Å.

## Related literature

For the biological activity and coordination ability of hydrazone compounds, see: Khattab (2005 ▶); Reiter *et al.* (1985 ▶). For the properties of isoxazole derivatives, see: Stevens & Albizati (1984 ▶). For examples of crystal structures of hydrazone compounds, see: Fun *et al.* (2008 ▶); Wei *et al.* (2009 ▶); Khaledi *et al.* (2008 ▶). For reference bond-length data, see: Allen *et al.* (1987 ▶).
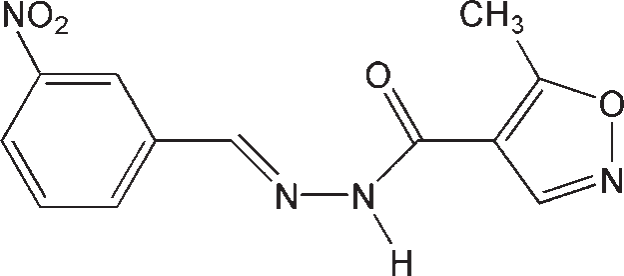


## Experimental

### 

#### Crystal data

C_12_H_10_N_4_O_4_
*M* *_r_* = 274.24
Monoclinic, 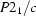

*a* = 4.8668 (8) Å
*b* = 25.202 (4) Å
*c* = 10.257 (2) Å
β = 100.721 (12)°
*V* = 1236.1 (4) Å^3^
*Z* = 4
Mo *K*α radiation
μ = 0.11 mm^−1^
*T* = 293 K
0.66 × 0.30 × 0.14 mm

#### Data collection

Bruker APEXII CCD area-detector diffractometer
Absorption correction: multi-scan (*SADABS*; Bruker, 2004 ▶) *T*_min_ = 0.951, *T*_max_ = 0.971
10436 measured reflections
2828 independent reflections
1943 reflections with *I* > 2σ(*I*)
*R*_int_ = 0.034

#### Refinement

*R*[*F* ^2^ > 2σ(*F* ^2^)] = 0.049
*wR*(*F* ^2^) = 0.129
*S* = 1.10
2828 reflections
186 parameters
1 restraint
H atoms treated by a mixture of independent and constrained refinement
Δρ_max_ = 0.32 e Å^−3^
Δρ_min_ = −0.24 e Å^−3^

### 

Data collection: *APEX2* (Bruker, 2004 ▶); cell refinement: *SAINT* (Bruker, 2004 ▶); data reduction: *SAINT*; program(s) used to solve structure: *SHELXS97* (Sheldrick, 2008 ▶); program(s) used to refine structure: *SHELXL97* (Sheldrick, 2008 ▶); molecular graphics: *SHELXTL* (Sheldrick, 2008 ▶); software used to prepare material for publication: *SHELXL97*.

## Supplementary Material

Crystal structure: contains datablocks General, I. DOI: 10.1107/S1600536809051733/rz2400sup1.cif

Structure factors: contains datablocks I. DOI: 10.1107/S1600536809051733/rz2400Isup2.hkl

Additional supplementary materials:  crystallographic information; 3D view; checkCIF report

## Figures and Tables

**Table 1:** Hydrogen-bond geometry (Å, °)

*D*—H⋯*A*	*D*—H	H⋯*A*	*D*⋯*A*	*D*—H⋯*A*
C3—H3*A*⋯N3	0.93	2.43	2.930 (2)	114
C12—H12⋯O4^i^	0.93	2.58	3.240 (2)	128
N2—H2⋯O2^ii^	0.90 (1)	1.95 (1)	2.855 (2)	179 (1)

Symmetry codes: (i) 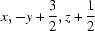; (ii) 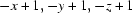.

**Fig. 1.**
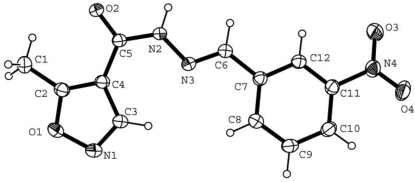
The molecular structure of of the title compound. Displacement ellipsoids are drawn at the 30% probability level.

**Fig. 2.**
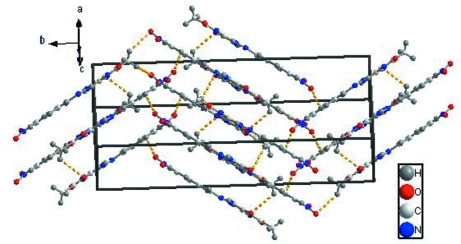
Packing diagram of the title compound. Intermolecular hydrogen bonds are shown as dashed lines.

## supplementary crystallographic information

### Comment

Hydrazone compounds have been widely studied because they exhibit extensive biological activities (Khattab, 2005) and coordination ability (Reiter *et al.*, 1985). Isoxazole compounds have also attracted much interest because of their fungicidal activity, plant-growth regulating activity and antibacterial activity (Stevens & Albizati, 1984). In the last few years, a large number of hydrazone derivatives have been reported (*e. g.* Fun *et al.*, 2008; Wei *et al.*, 2009; Khaledi *et al.*, 2008). In order to study the properties of new compounds containing both the hydrazine and isoxazole groups, the title compound has been synthesized and its crystal structure is reported herein.

The molecule of the title compound (Fig. 1) exhibits an *E* configuration with respect to the C6═N3 double bond, with the C7—C6—N3—N2 torsion angle of 178.8 (2)°. Bond lengths (Allen *et al.*, 1987) and angles in the molecule are within normal ranges. The molecule is approximately planar [maximum displacement 0.1740 (17) Å for atom O4], with a dihedral angle between the benzene and isoxazole rings of 1.36 (5)°. The molecular conformation is enforced by an intramolecular C—H···N hydrogen bond. In the crystal packing, centrosymmetrically related molecules are connected into dimers by N—H···O hydrogen bonds (Table 1). The dimers are further linked into a three-dimensional network (Fig. 2) by intermolecular C—H···O hydrogen bonds and by π···π stacking interactions involving adjacent benzene and isoxazole rings, with centroid-to-centroid separations of 3.861 (3) Å.

### Experimental

The title compound, C_12_H_10_N_4_O_4_, was synthesized as follows: 3-nitrobenzaldehyde (2.2 g) and 5-methylisoxazole-4-carbonyl hydrazine (2.0 g, 0.014 mol) were mixed with glacial acetic acid (50 ml). The mixture was heated at 65 for 4 h, then the precipitate was collected by filtration and washed with water, chloroform and ethanol. The product was recrystallized from ethanol, then dried under reduced pressure. The title compound was obtained with a yield of 72.5%. Colourless block-shaped crystals suitable for X-ray analysis were obtained by slow evaporation of a dimethylformamide solution.

### Refinement

The H atom bound to the N2 atom was located in a difference Fourier map and refined freely with the N–H distance restrained to 0.90 Å. All other H atoms were positioned geometrically and allowed to ride on their parent atoms, with C—H = 0.93–0.96 Å, and with *U*_iso_ = 1.2 *U*_iso_(C) or 1.5 *U*_iso_(C) for methyl H atoms.

### Crystal data

**Table:**

C_12_H_10_N_4_O_4_	*F*(000) = 568
*M**_r_* = 274.24	*D*_x_ = 1.474 Mg m^−^^3^
Monoclinic, *P*2_1_/*c*	Mo *K*α radiation, λ = 0.71073 Å
Hall symbol: -P 2ybc	Cell parameters from 2571 reflections
*a* = 4.8668 (8) Å	θ = 2.6–23.7°
*b* = 25.202 (4) Å	µ = 0.11 mm^−^^1^
*c* = 10.257 (2) Å	*T* = 293 K
β = 100.721 (12)°	Block, colourless
*V* = 1236.1 (4) Å^3^	0.66 × 0.30 × 0.14 mm
*Z* = 4	

### Data collection

**Table:**

Bruker APEXII CCD area-detector diffractometer	2828 independent reflections
Radiation source: sealed tube	1943 reflections with *I* > 2σ(*I*)
graphite	*R*_int_ = 0.034
phi and ω scans	θ_max_ = 27.6°, θ_min_ = 2.2°
Absorption correction: multi-scan (*SADABS*; Bruker, 2004)	*h* = −5→6
*T*_min_ = 0.951, *T*_max_ = 0.971	*k* = −32→30
10436 measured reflections	*l* = −13→12

### Refinement

**Table:**

Refinement on *F*^2^	Primary atom site location: structure-invariant direct methods
Least-squares matrix: full	Secondary atom site location: difference Fourier map
*R*[*F*^2^ > 2σ(*F*^2^)] = 0.049	Hydrogen site location: inferred from neighbouring sites
*wR*(*F*^2^) = 0.129	H atoms treated by a mixture of independent and constrained refinement
*S* = 1.10	*w* = 1/[σ^2^(*F*_o_^2^) + (0.054*P*)^2^ + 0.2908*P*] where *P* = (*F*_o_^2^ + 2*F*_c_^2^)/3
2828 reflections	(Δ/σ)_max_ = 0.012
186 parameters	Δρ_max_ = 0.32 e Å^−^^3^
1 restraint	Δρ_min_ = −0.24 e Å^−^^3^

### Special details

**Table:**

Geometry. All e.s.d.'s (except the e.s.d. in the dihedral angle between two l.s. planes) are estimated using the full covariance matrix. The cell e.s.d.'s are taken into account individually in the estimation of e.s.d.'s in distances, angles and torsion angles; correlations between e.s.d.'s in cell parameters are only used when they are defined by crystal symmetry. An approximate (isotropic) treatment of cell e.s.d.'s is used for estimating e.s.d.'s involving l.s. planes.
Refinement. Refinement of *F*^2^ against ALL reflections. The weighted *R*-factor *wR* and goodness of fit *S* are based on *F*^2^, conventional *R*-factors *R* are based on *F*, with *F* set to zero for negative *F*^2^. The threshold expression of *F*^2^ > σ(*F*^2^) is used only for calculating *R*-factors(gt) *etc*. and is not relevant to the choice of reflections for refinement. *R*-factors based on *F*^2^ are statistically about twice as large as those based on *F*, and *R*- factors based on ALL data will be even larger.

### Fractional atomic coordinates and isotropic or equivalent isotropic displacement parameters (Å^2^)

**Table:**

	*x*	*y*	*z*	*U*_iso_*/*U*_eq_	
C1	−0.2323 (5)	0.37462 (8)	0.4047 (2)	0.0542 (5)	
H1A	−0.4117	0.3672	0.4267	0.081*	
H1B	−0.1073	0.3871	0.4823	0.081*	
H1C	−0.1581	0.3429	0.3729	0.081*	
C2	−0.2629 (4)	0.41576 (7)	0.30054 (17)	0.0410 (4)	
C3	−0.2693 (4)	0.47950 (7)	0.15576 (17)	0.0457 (5)	
H3A	−0.2179	0.5097	0.1142	0.055*	
C4	−0.1173 (4)	0.45970 (7)	0.27849 (16)	0.0378 (4)	
C5	0.1366 (4)	0.47775 (7)	0.36822 (16)	0.0367 (4)	
C6	0.3416 (4)	0.59572 (7)	0.23565 (16)	0.0415 (5)	
H6	0.4994	0.5997	0.3017	0.050*	
C7	0.2862 (4)	0.63497 (7)	0.12970 (16)	0.0377 (4)	
C8	0.0622 (4)	0.63082 (7)	0.02243 (17)	0.0438 (5)	
H8	−0.0627	0.6027	0.0188	0.053*	
C9	0.0253 (4)	0.66798 (8)	−0.07759 (18)	0.0500 (5)	
H9	−0.1237	0.6646	−0.1485	0.060*	
C10	0.2072 (4)	0.71022 (7)	−0.07375 (17)	0.0471 (5)	
H10	0.1832	0.7354	−0.1412	0.057*	
C11	0.4254 (4)	0.71402 (7)	0.03287 (16)	0.0405 (4)	
C12	0.4682 (4)	0.67752 (7)	0.13459 (16)	0.0404 (4)	
H12	0.6168	0.6814	0.2054	0.049*	
H2	0.426 (3)	0.5310 (8)	0.4040 (16)	0.053 (6)*	
N1	−0.4859 (4)	0.45108 (7)	0.10830 (15)	0.0544 (5)	
N2	0.2682 (3)	0.52281 (6)	0.34603 (13)	0.0406 (4)	
N3	0.1809 (3)	0.55618 (6)	0.24086 (13)	0.0395 (4)	
N4	0.6224 (4)	0.75843 (6)	0.03927 (15)	0.0503 (4)	
O1	−0.4845 (3)	0.40961 (5)	0.20178 (13)	0.0512 (4)	
O2	0.2363 (3)	0.45160 (5)	0.46820 (11)	0.0462 (4)	
O3	0.7959 (4)	0.76447 (7)	0.14015 (15)	0.0811 (6)	
O4	0.6048 (4)	0.78753 (6)	−0.05611 (14)	0.0689 (5)	

### Atomic displacement parameters (Å^2^)

**Table:**

	*U*^11^	*U*^22^	*U*^33^	*U*^12^	*U*^13^	*U*^23^
C1	0.0540 (14)	0.0443 (11)	0.0618 (12)	−0.0070 (9)	0.0046 (10)	0.0082 (9)
C2	0.0369 (11)	0.0402 (9)	0.0438 (9)	0.0012 (8)	0.0017 (8)	−0.0035 (7)
C3	0.0446 (12)	0.0445 (10)	0.0426 (9)	−0.0046 (8)	−0.0061 (8)	0.0023 (8)
C4	0.0351 (10)	0.0382 (9)	0.0377 (8)	0.0008 (7)	0.0009 (7)	−0.0005 (7)
C5	0.0345 (10)	0.0374 (9)	0.0361 (8)	0.0010 (7)	0.0013 (7)	0.0012 (7)
C6	0.0413 (12)	0.0424 (10)	0.0378 (8)	−0.0047 (8)	−0.0006 (8)	0.0023 (7)
C7	0.0405 (11)	0.0370 (9)	0.0351 (8)	0.0000 (7)	0.0058 (7)	−0.0003 (7)
C8	0.0433 (12)	0.0427 (10)	0.0431 (9)	−0.0045 (8)	0.0018 (8)	−0.0004 (7)
C9	0.0503 (13)	0.0523 (11)	0.0417 (9)	−0.0012 (9)	−0.0061 (9)	0.0032 (8)
C10	0.0554 (14)	0.0451 (10)	0.0389 (9)	0.0026 (9)	0.0037 (9)	0.0055 (8)
C11	0.0492 (12)	0.0344 (9)	0.0380 (8)	−0.0025 (8)	0.0087 (8)	−0.0026 (7)
C12	0.0452 (11)	0.0407 (9)	0.0335 (8)	−0.0032 (8)	0.0023 (8)	−0.0013 (7)
N1	0.0526 (11)	0.0535 (10)	0.0494 (9)	−0.0058 (8)	−0.0108 (8)	0.0045 (7)
N2	0.0383 (9)	0.0419 (8)	0.0369 (7)	−0.0062 (7)	−0.0052 (7)	0.0070 (6)
N3	0.0408 (9)	0.0392 (8)	0.0361 (7)	−0.0012 (7)	0.0005 (6)	0.0045 (6)
N4	0.0657 (13)	0.0404 (9)	0.0448 (8)	−0.0076 (8)	0.0105 (8)	−0.0011 (7)
O1	0.0437 (9)	0.0487 (7)	0.0558 (8)	−0.0099 (6)	−0.0047 (6)	−0.0022 (6)
O2	0.0435 (8)	0.0460 (7)	0.0436 (6)	−0.0060 (6)	−0.0065 (6)	0.0122 (5)
O3	0.1020 (15)	0.0733 (11)	0.0575 (9)	−0.0453 (10)	−0.0124 (9)	0.0048 (8)
O4	0.0948 (14)	0.0547 (9)	0.0567 (8)	−0.0164 (8)	0.0131 (8)	0.0157 (7)

### Geometric parameters (Å, °)

**Table:**

C1—C2	1.476 (2)	C7—C8	1.401 (2)
C1—H1A	0.9600	C8—C9	1.376 (3)
C1—H1B	0.9600	C8—H8	0.9300
C1—H1C	0.9600	C9—C10	1.381 (3)
C2—O1	1.344 (2)	C9—H9	0.9300
C2—C4	1.356 (3)	C10—C11	1.379 (3)
C3—N1	1.292 (2)	C10—H10	0.9300
C3—C4	1.426 (2)	C11—C12	1.377 (2)
C3—H3A	0.9300	C11—N4	1.468 (2)
C4—C5	1.469 (2)	C12—H12	0.9300
C5—O2	1.2394 (19)	N1—O1	1.417 (2)
C5—N2	1.344 (2)	N2—N3	1.3714 (19)
C6—N3	1.274 (2)	N2—H2	0.901 (10)
C6—C7	1.457 (2)	N4—O4	1.213 (2)
C6—H6	0.9300	N4—O3	1.217 (2)
C7—C12	1.386 (2)		
			
C2—C1—H1A	109.5	C9—C8—C7	120.61 (18)
C2—C1—H1B	109.5	C9—C8—H8	119.7
H1A—C1—H1B	109.5	C7—C8—H8	119.7
C2—C1—H1C	109.5	C8—C9—C10	120.63 (17)
H1A—C1—H1C	109.5	C8—C9—H9	119.7
H1B—C1—H1C	109.5	C10—C9—H9	119.7
O1—C2—C4	109.83 (15)	C11—C10—C9	118.12 (16)
O1—C2—C1	115.04 (16)	C11—C10—H10	120.9
C4—C2—C1	135.13 (16)	C9—C10—H10	120.9
N1—C3—C4	113.03 (17)	C12—C11—C10	122.69 (17)
N1—C3—H3A	123.5	C12—C11—N4	118.03 (16)
C4—C3—H3A	123.5	C10—C11—N4	119.27 (15)
C2—C4—C3	103.39 (15)	C11—C12—C7	118.94 (16)
C2—C4—C5	123.58 (15)	C11—C12—H12	120.5
C3—C4—C5	133.03 (16)	C7—C12—H12	120.5
O2—C5—N2	117.65 (15)	C3—N1—O1	104.65 (14)
O2—C5—C4	120.52 (16)	C5—N2—N3	124.32 (14)
N2—C5—C4	121.83 (14)	C5—N2—H2	117.0 (13)
N3—C6—C7	122.18 (16)	N3—N2—H2	118.7 (13)
N3—C6—H6	118.9	C6—N3—N2	114.20 (14)
C7—C6—H6	118.9	O4—N4—O3	122.94 (17)
C12—C7—C8	119.00 (15)	O4—N4—C11	118.53 (16)
C12—C7—C6	118.04 (15)	O3—N4—C11	118.53 (15)
C8—C7—C6	122.94 (16)	C2—O1—N1	109.09 (14)
			
O1—C2—C4—C3	−0.7 (2)	C9—C10—C11—N4	179.80 (18)
C1—C2—C4—C3	179.0 (2)	C10—C11—C12—C7	0.4 (3)
O1—C2—C4—C5	179.85 (17)	N4—C11—C12—C7	−179.30 (17)
C1—C2—C4—C5	−0.5 (4)	C8—C7—C12—C11	−0.9 (3)
N1—C3—C4—C2	0.5 (2)	C6—C7—C12—C11	177.65 (17)
N1—C3—C4—C5	179.9 (2)	C4—C3—N1—O1	−0.1 (2)
C2—C4—C5—O2	2.7 (3)	O2—C5—N2—N3	−179.10 (16)
C3—C4—C5—O2	−176.56 (19)	C4—C5—N2—N3	0.6 (3)
C2—C4—C5—N2	−176.99 (18)	C7—C6—N3—N2	178.84 (16)
C3—C4—C5—N2	3.7 (3)	C5—N2—N3—C6	−177.51 (18)
N3—C6—C7—C12	178.03 (18)	C12—C11—N4—O4	172.21 (18)
N3—C6—C7—C8	−3.5 (3)	C10—C11—N4—O4	−7.5 (3)
C12—C7—C8—C9	0.9 (3)	C12—C11—N4—O3	−7.9 (3)
C6—C7—C8—C9	−177.58 (19)	C10—C11—N4—O3	172.4 (2)
C7—C8—C9—C10	−0.4 (3)	C4—C2—O1—N1	0.6 (2)
C8—C9—C10—C11	−0.1 (3)	C1—C2—O1—N1	−179.08 (16)
C9—C10—C11—C12	0.1 (3)	C3—N1—O1—C2	−0.3 (2)

### Hydrogen-bond geometry (Å, °)

**Table:**

*D*—H···*A*	*D*—H	H···*A*	*D*···*A*	*D*—H···*A*
C3—H3A···N3	0.93	2.43	2.930 (2)	114
C12—H12···O4^i^	0.93	2.58	3.240 (2)	128
N2—H2···O2^ii^	0.90 (1)	1.95 (1)	2.855 (2)	179 (1)

Symmetry codes: (i) *x*, −*y*+3/2, *z*+1/2; (ii) −*x*+1, −*y*+1, −*z*+1.
